# Congenital Heart Defects and Ciliopathies Associated With Renal Phenotypes

**DOI:** 10.3389/fped.2018.00175

**Published:** 2018-06-15

**Authors:** George C. Gabriel, Gregory J. Pazour, Cecilia W. Lo

**Affiliations:** ^1^Department of Developmental Biology, University of Pittsburgh School of Medicine, Pittsburgh, PA, United States; ^2^Program in Molecular Medicine, University of Massachusetts Medical School, Worcester, MA, United States

**Keywords:** cilia, CAKUT, congenital heart disease, congenital abnormalities, ciliopathies, genetic syndromes

## Abstract

Congenital heart disease (CHD) is one of the most common birth defects, and recent studies indicate cilia-related mutations play a central role in the genetic etiology of CHD. As cilia are also known to have important roles in kidney development and disease, it is not surprising that renal anomalies were found to be enriched among CHD mutant mice recovered in a large-scale mouse forward genetic screen. Indeed 42% of mutations identified to cause both CHD and renal anomalies were cilia-related. Many of these cilia mutations comprise cilia transition zone or inversin compartment components, consistent with the known role of these cilia proteins in a wide variety of ciliopathies. The high prevalence of CHD with congenital anomalies of the kidney and urinary tract (CAKUT) observed in mice was also corroborated with clinical studies that showed 20–30% of CHD patients have renal anomalies. Together these findings suggest CHD patients may benefit from early screening for renal anomalies to allow early diagnosis and intervention to improve outcome for this vulnerable patient population.

Congenital heart disease (CHD) is the most common birth defect, occurring in up to 1% of live births (1). Interestingly, CHD often presents in combination with extra cardiac defects including congenital anomalies of the kidney and urinary tract (CAKUT) (2–4). In fact, renal or urinary system defects are associated with 23.1% of congenital heart defects (5). These findings suggest an overlapping genetic etiology for CHD and CAKUT. Consistent with this is the fact that several previously reported mouse models (not individually discussed in this review) have found genetic disruption of a single gene can affect both heart and kidney development, and that many genetic syndromes can present with both cardiac and renal anomalies (6–9). Hence, insights into the genetic etiology of CHD may help elucidate the etiology of CAKUT. Relevant to this is the recent unexpected finding from a large-scale forward genetic screen in mice indicating a significant role for cilia-related mutations in the pathogenesis of CHD (10). As cilia mutations are well-described in the context of CAKUT, this would suggest cilia defects may have a central role in mediating both CHD and CAKUT phenotypes. Below, we briefly discuss CAKUT and its association with CHD, and its shared genetic etiology involving cilia-related mutations.

## Association of heart and kidney phenotypes in genetic syndromes

CAKUT represent a broad range of kidney and urinary tract defects. This can include abnormalities in the shape, size, or structure of the kidney including kidney agenesis, kidney hypoplasia or dysplasia, horseshoe/fused kidney, cystic kidneys, or duplex/multiplex kidney, and multiple collecting ducts or ureters (11). These defects can occur in combination such as with the finding of cystic dysplastic kidneys. These structural abnormalities can result in vesicoureteral reflux, ureteropelvic junction obstruction, hydroureter, and hydronephrosis (10, 12). In humans, monogenic mutations in several genes have been implicated in CAKUT development; however, these mutations explain only 5–20% of patient disease (13). Additional causes of CAKUT phenotypes include genetic syndromes, which can present with a spectrum of phenotypes often involving defects in many organs including both the heart and the kidney.

In DiGeorge syndrome, heterozygous deletion of chromosome 22q11.2 is associated with multiple organ defects, including the heart, the kidney, the thymus, and the nervous system. It has an estimated prevalence of 1:3,000 to 1:4,000 live births, though prenatal chromosomal analysis suggests this may be an underestimate, and affected patients characteristically present with CHD, thymus hypoplasia or agenesis, and craniofacial defects (14–16). Approximately 20–30% of DiGeorge syndrome patients also exhibit CAKUT (17). Haploinsufficiency of *TBX1*, a transcription factor within the T-box family, has been shown to contribute to the cardiac phenotypes associated with this disorder (18, 19). More recently, another gene within the 22q11.2 locus, *CRKL*, an adaptor protein that regulates tyrosine kinase signaling, was found to contribute to developmental kidney defects in the context of DiGeorge syndrome (20, 21).

Another syndrome known as VACTERL association also presents with both congenital heart defects and renal abnormalities. Occurring in 1:10,000 to 1:40,000 live births, VACTERL association is a birth defect associated with at least 3 of the following phenotypes: vertebral defects, anal atresia, cardiac defects, trachea-esophageal fistula, renal defects, and limb defects (22). In a large study of patients diagnosed with VACTERL association, 80% exhibited kidney defects and 48% exhibited cardiac defects (23). In humans, several genes have been related to VACTERL association including *FGF8, FOXF1, HOXD13, LPP, TRAP1, and ZIC3*, all of which have been implicated in kidney developmental anomalies, with ZIC3 being an X-linked gene also shown to cause heterotaxy (24).

A rarer genetic disorder known as Fraser syndrome can also cause both CHD and genitourinary abnormalities. It is a recessive disorder with a prevalence of only 1 in 200,000 (25). Patients with Fraser syndrome can present with eye defects, syndactyly, kidney defects, most commonly kidney agenesis, and heart defects (25). Fraser syndrome is associated with mutations in *FRAS1, FREM2*, or *GRIP1*, all of which are known to interact to form a FRAS/FREM complex that is localized to the basement membrane (26, 27). Mutations in these genes cause loss of the FRAS/FREM complex that likely drives the disease phenotypes observed in Fraser syndrome, although the precise disease mechanism remains unclear (27, 28).

## Cilia and the genetic landscape of CHD

The close association of renal anomalies with CHD suggests insights into the genetics of CHD can yield significant insights into the mechanism of disease pathogenesis for CAKUT. Interestingly, the pursuit of a large-scale forward genetic screen in mice for mutations causing CHD yielded a large preponderance of mutations in cilia-related genes (29). From screening more than 100,000 ethylnitrosourea (ENU) mutagenized mice using *in utero* fetal ultrasound phenotyping, we recovered over 200 mouse lines with a wide spectrum of CHD. Using whole exome sequencing nearly 100 CHD-causing mutations were recovered in 61 genes, 34 (55.7%) of which were cilia-related (29). As this is a phenotype driven mutagenesis screen in which fetal echocardiography was used to identify mutants with CHD, these findings point to cilia as playing a central role in the pathogenesis of CHD. This is corroborated with the completion of the screen, showing over 50 genes are cilia-related among 100 genes recovered causing CHD. Moreover, analysis of de novo mutations identified in whole exome sequencing analysis from CHD patients also identified similar mutations contributing to CHD pathogenesis (30).

## Cilia and ciliopathies in CHD, renal anomalies, and other human diseases

**Figure 1:**
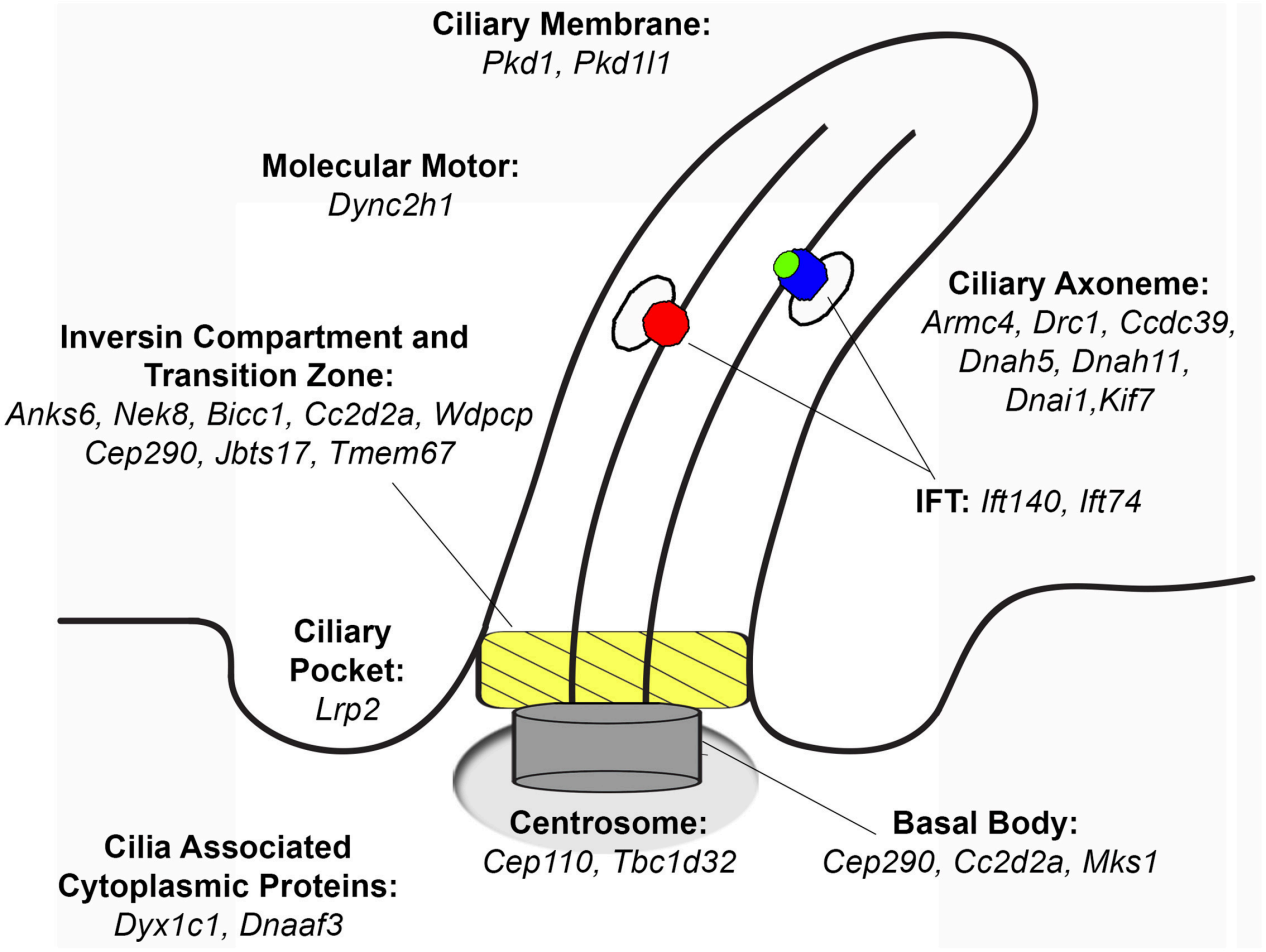
Ciliary localization of proteins that when mutated cause congenital heart disease recovered from a large-scale screen. Mutations in several of these proteins can also cause CAKUT. Adapted from Damerla et al. (31).

Cilia are microtubule-based organelles that have well-described compartments including an axoneme that projects from the apical cell surface, and a basal body base with an overlying transition zone and inversin compartment that regulate protein trafficking into and out of the cilium (Figure 1) (32). Cilia can be motile with the expression of motor dyneins that drive ciliary motility, or nonmotile, termed primary cilia, which extend from almost all cell types in the body, including cells of the developing heart, and are known to serve important cell signaling functions by mediating various signal transduction processes (33–35). Motile cilia are required for left-right patterning as well as in mediating sperm motility, mucociliary clearance in the airway, and also cerebral spinal fluid flow in the brain. Importantly, it is now appreciated that cilia mutations can cause a wide spectrum of human diseases collectively known as ciliopathies (31). Many of these ciliopathies can present with renal anomalies, such as in nephronepthesis, Meckel-Gruber syndrome (MKS), Bardet-Biedl syndrome (BBS), Joubert syndrome, Alstrom syndrome and polycystic kidney disease among others (36). The renal defects observed may include CAKUT, polycystic kidney disease or progressive renal dysfunction. Other structural birth defects observed in the ciliopathies include CHD, cardiomyopathy, skeletal malformations, brain abnormalities, blindness, and obesity (31).

Many of the mutations known to cause renal anomalies within the ciliopathy spectrum are associated with proteins localized in the cilia transition zone or inversin compartment. Thus, among the 61 genes recovered causing CHD, half (*N* = 34) were cilia-related, including 11 affecting transition zone or inversin compartment components (29). While these are mainly thought to disrupt primary cilia function, recent studies showed ciliopathies generally considered to involve primary cilia defects may also impact motile cilia function. This is indicated by the observation of airway clearance defects in patients diagnosed with ciliopathies such as Leber congenital amaurosis or Sensenbrenner syndrome (37, 38). This is not unexpected given over 70% of proteins required for primary cilia function are also expressed in motile cilia (39). Indeed, airway clearance defects are the hallmark of a ciliopathy known as primary ciliary dyskinesia (PCD).

PCD patients have severe sinopulmonary disease and typically this is associated with mutations in genes required for motile cilia function, such as motor dyneins (40–42). It is interesting to note some isolated reports of bronchiectasis associated with polycystic kidney disease, although the connection with airway clearance defects is unknown (43). PCD patients also can exhibit laterality defects, a reflection of the requirement for motile cilia in the specification of the left-right body axis (44, 45). Consistent with this, PCD patients can display a spectrum of phenotypes including situs solitus (normal visceral organ situs), situs inversus totalis also known as Kartagener's syndrome (reversed visceral organ situs), and heterotaxy, or the randomization of visceral organ situs. In this context, CHD is often associated with heterotaxy as a result of defects in motile cilia function.

Interestingly, some ciliopathies thought to affect only primary cilia function also can be associated with laterality defects, one prominent example being MKS, a ciliopathy that causes severe birth defects and is usually prenatal or neonatal lethal. Studies of *Mks1* mutant mice showed defects involving not only the primary cilia, but also laterality defects associated with the loss of motile cilia function in the embryonic node (46). Together, these findings suggest that the distinction of ciliopathies as either affecting motile or primary cilia can be problematic, given the promiscuity of cilia genes in having functional roles in both primary and motile cilia.

Intriguingly, primary cilia proteins can be found in other cellular locations, suggesting that they may have other non-ciliary functions (47). In fact, ciliary proteins are also involved in a wide range of cellular processes including cell cycle progression, cytoskeletal organization, and vesicular trafficking (48–50). Further, cilia themselves are involved in several cell signaling pathways including the sonic hedgehog, TGFβ, wnt, and notch pathways, and defects can arise in these pathways through defective cilia or pathway components outside of the cilium (51). Thus, future research will be important to determine the precise roles cilia and cilia associated proteins play in CHD and CAKUT development.

## Mutations causing CHD and CAKUT phenotypes

**Figure 2:**
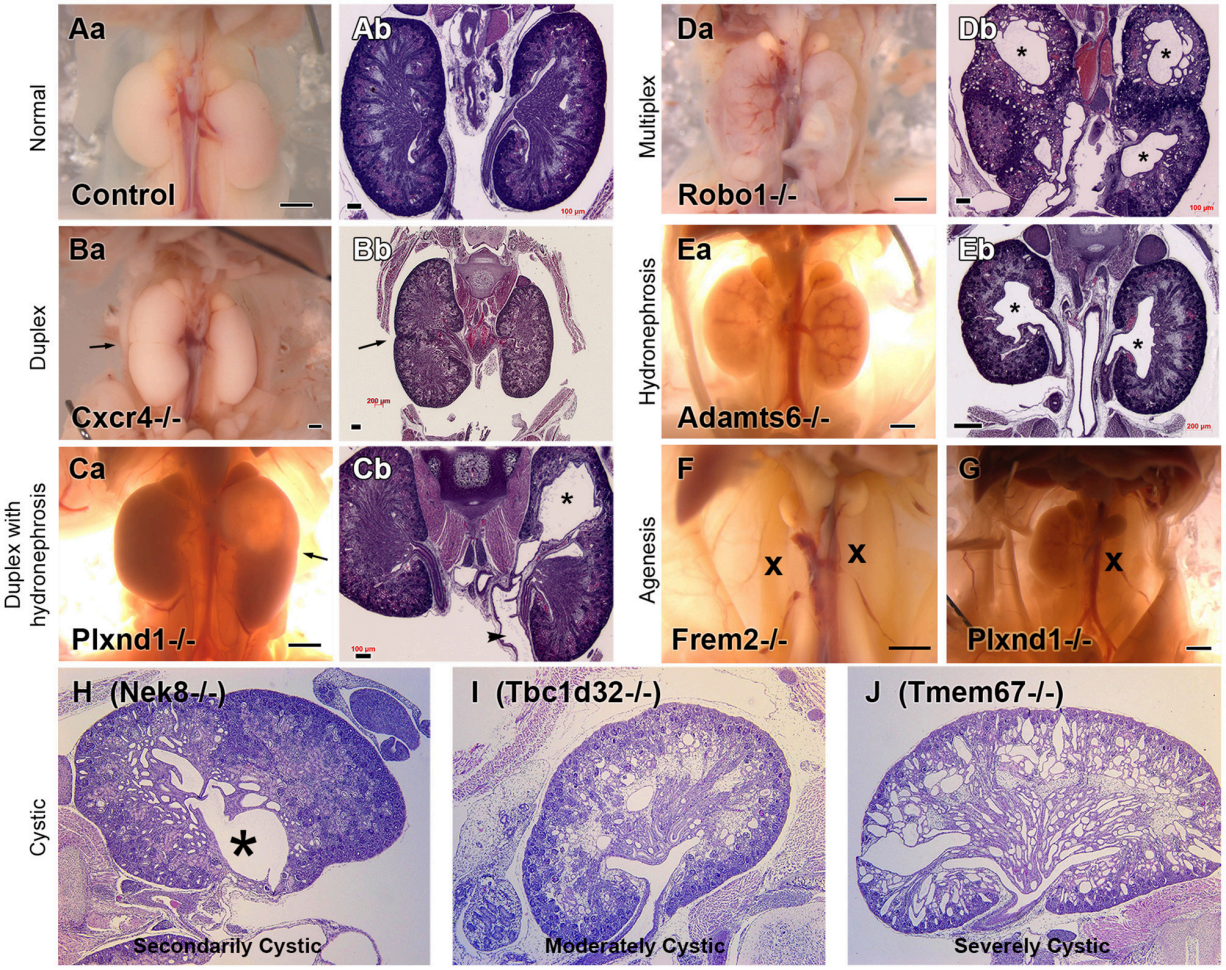
Kidney Phenotypes Observed in Mice with Congenital Heart Disease. Representative examples of the kidney phenotypes observed in mice with congenital heart disease recovered from a large scale forward genetic screen. Phenotypes can be observed at necropsy and upon H&E staining. Adapted from San Agustin et al. (10). Control necropsy **(Aa)** and control H&E stained kidneys **(Ab)** can be compared with mutant necropsy **(Ba,Ca,Da,Ea,F,G)** and mutant H&E stained kidneys **(Bb,Cb,Db,Eb, H–J)**. ^*^represents hydronephrosis.

Extra-cardiac phenotypes, including renal anomalies, were recovered in many CHD mutant mice even though the screen was entirely focused on CHD phenotyping. This included a spectrum of kidney phenotypes including duplex kidney, multiplex kidney, hydronephrosis, kidney agenesis, and cystic kidney (Figure 2), with the most commonly observed renal phenotype being duplex kidneys (10). Among the 39 mutant mouse lines recovered with congenital kidney abnormalities, the most common CHD phenotype is double outlet right ventricle (DORV), occurring in 19/39 lines (48.7%). This is followed by atrioventricular septal defect, which occurred in 15/39 lines (38.5%; Table 1). Other CHD phenotypes associated with kidney defects included transposition of the great arteries (TGA), persistent truncus arteriosus (PTA), ventricular septal defect (VSD), aortic arch anomalies, and biventricular hypertrophy (Table 1).

**Table 1:** Renal anomalies associated with genes causing congenital heart defects.

**Gene^a^**	**Duplex/multiplex**	**Hydro-nephrosis**	**Cystic**	**Agenesis/Hypoplasia**	**Cardiac phenotype**
Anks6^*^ (10)	–	–	80%	–	DORV, TGA, AVSD, VSD
Bicc1^*^ (8)	–	–	100%	–	DORV, TGA, AVSD, IAA
Cc2d2a^*^ (5)	–	–	100%	–	TGA, PTA, DORV, AVSD, Pulmonary Atresia
***Cep110* (5)**	–	60%	–	–	DORV, AVSD, VSD
Cep290^*^ (13)	15%	–	100%	–	DORV, AVSD
Dync2h1^*^ (6)	50%	–	–	–	PTA/Pulmonary atresia, VSD, MAPCA
***Lrp2* (3)**	–	100%	100%	–	PTA, VSD, Ao arch defects
***Pkd1*** **(4)**	–	–	100%	–	Biventricular Hypertrophy
***Ptk7*** **(3)**	–	–	100%	–	DORV, VSD, Biventricular Hypertrophy
Tbc1d32^*^ (12)	25%	–	92%	–	PTA, DORV, TGA, AVSD
Tmem67^*^ (12)	42%	–	100%	–	DORV, AVSD, IAA
*Adamts6* (16)	–	56%	–	–	DORV, OA
Ap1b1^*^ (10)	–	–	80%	–	AVSD, VSD, Atrial Isomerism
*Ap2b1* (3)	–	–	75%	–	DORV/Taussig-Bing, AVSD, VSD, Ao arch defects
*Bmp10* (2)	–	–	100%	–	DORV
*Cxcr4* (5)	60%	–	–	–	VSD, Ao atresia, Ao arch defects
*Frem2* (10)	–	–	–	100%	Biventricular Hypertrophy
*Lama5* (5)	–	–	–	60%	DORV
*Plxnd1* (3)	–	–	–	63%	PTA, DORV, AVSD, RAA
*Prdm1* (8)	38%	–	–	–	DORV, AVSD, VSD, Ao atresia, Ao arch defects
*Qrich1* (3)	–	–	–	67%	DORV, AVSD, noncompaction
*Robo1* (5)	100% (M)	–	–	–	VSD, Biventricular Hypertrophy
*Slit2* (7)	100% (M)	–	–	–	DORV, VSD, Ao arch defects
*Snx17* (3)	66%	–	–	–	DORV/OA, AVSD
*Wnt5a* (7)	43%	–	–	–	DORV, PTA, AVSD, VSD
*Zbtb14* (4)	–	–	100%	–	DORV, AVSD, atrioventricular and semilunar valve defects

a *Gene with dark highlighting are ciliary components based on proteomic and other studies*.

Gene names followed by

* *indicate mutations that cause situs defects*.

*Parenthesis indicate the number of mutants analyzed*.

*Adapted from San Agustin et al. (10)*.

**Figure 3:**
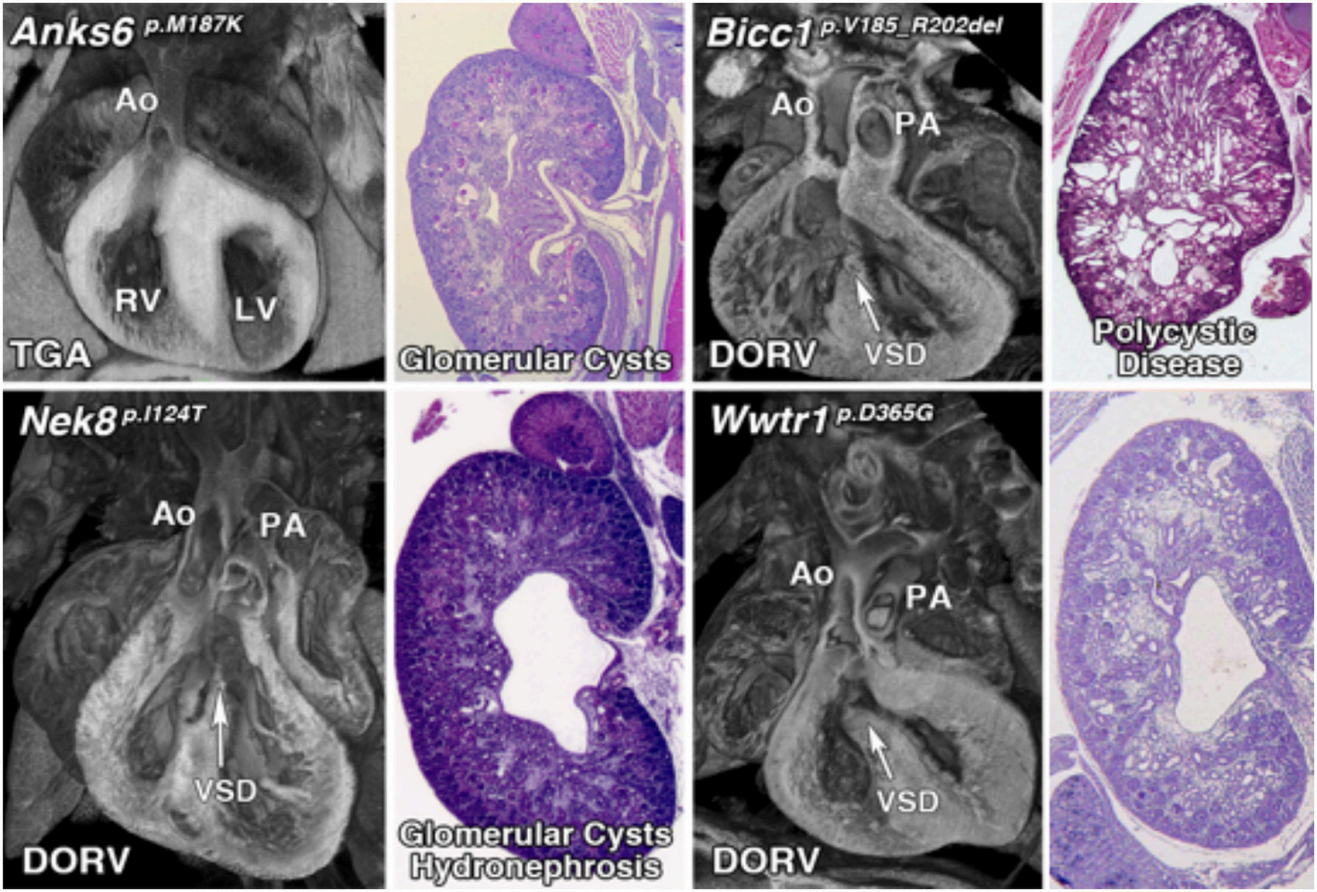
Mutations in interacting proteins cause a similar spectrum of both heart and kidney phenotypes. Mutations in four interacting proteins, *Anks6, Bicc1, Nek8*, and *Wwtr1* cause similar phenotypes including both congenital heart disease and cystic kidney disease (Unpublished data).

Previous attempts to recover CAKUT genes have been confounded by incomplete penetrance and the probable involvement of more complex genetics. The few CAKUT genes previously identified are largely those that mediate the early steps in kidney development and patterning. Other than *Pax2*, which causes renal coloboma syndrome, and *Hnf1b*, which causes renal cystic disease and diabetes, most genes identified to cause CAKUT are associated with disease in only a few patients and hence, the evidence supporting pathogenesis is limited (52). Among the 135 CHD mouse lines recovered, 39 (28.8%) had kidney defects caused by mutations in 26 genes, 11 (42%) being known cilia genes (Table 1, Figure 1) (10). Remarkably, this included four cilia genes that encoded known direct protein-protein interactors: *Anks6, Nek8, Bicc1*, and *Wwtr1* (53–56). These mutations were found in four independent CHD mouse lines, with mutation in each gene causing a similar spectrum of kidney and heart defect phenotypes comprising double outlet right ventricle and cystic kidney disease (Figure 3).

## Clinical study showing association of CHD with CAKUT phenotypes

**Figure 4:**
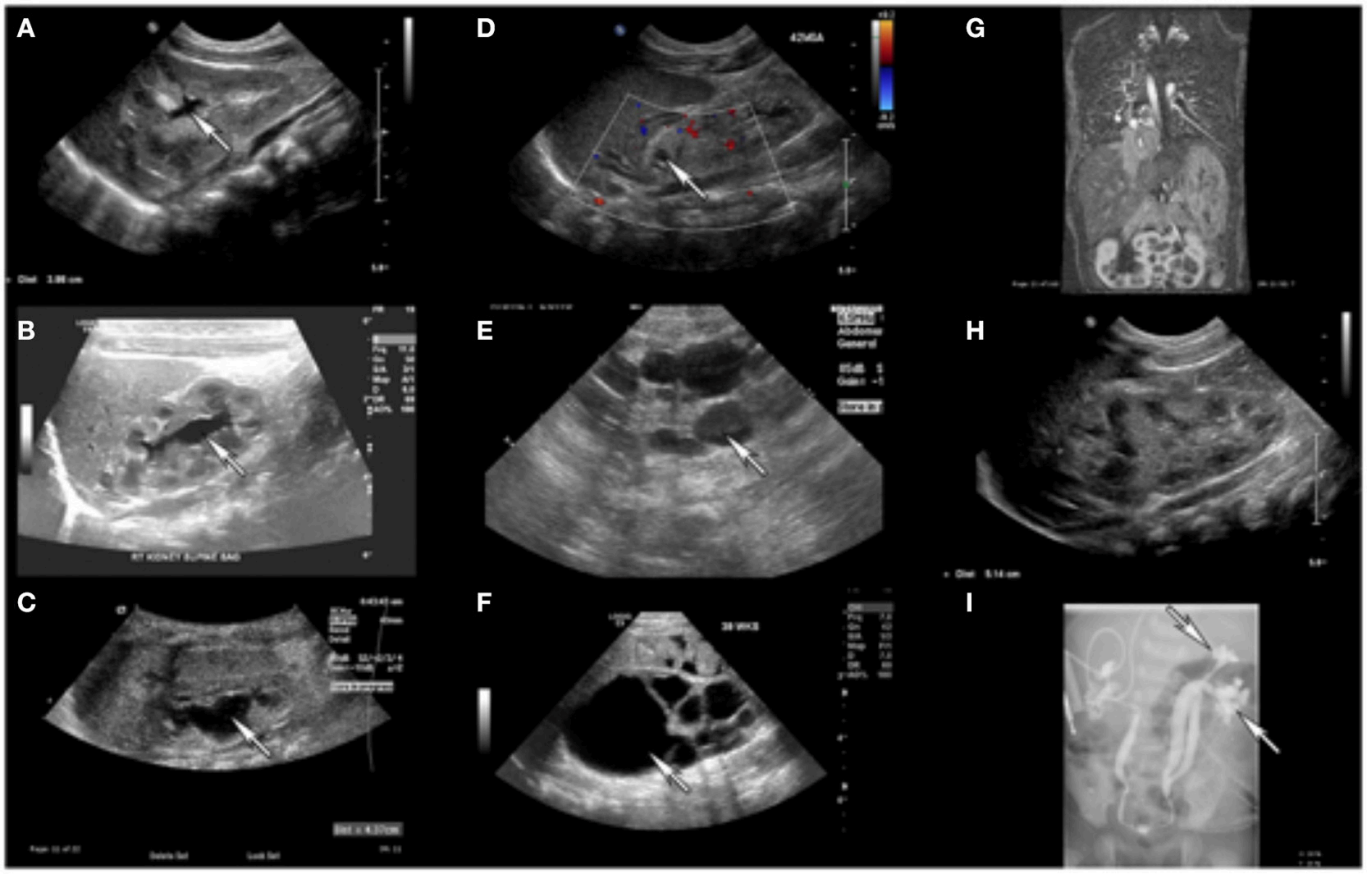
Kidney Defects Observed in Patients with CHD. Renal ultrasound of patients with congenital heart disease unveils a spectrum of renal abnormalities including intrarenal collecting system dilation **(A–C)**, cystic-dysplastic abnormalities **(D–F)**, horseshoe kidney **(G)**, and duplicated collecting system depicted with renal ultrasound **(H)**, and voiding cystourethrogram **(I)**. Adapted from San Agustin et al. (10).

The relevance of CAKUT abnormalities in CHD was confirmed with a clinical study of 77 CHD patients recruited from the Children's Hospital of Pittsburgh. Retrospective review of the medical records of these 77 patients revealed 23 (30%) also had some form of renal defect such as renal cysts, kidney agenesis, cystic dysplastic kidneys, and horseshoe kidney (Figure 4, Table 2) (10). These findings are in agreement with a previous epidemiological study in the Atlanta metro area, which showed 23% of 8,000 subjects with CHD also had renal abnormalities (1). Thus, both the mouse and human studies point to a significant overlap in the genetic etiology of CHD and kidney abnormalities. This probably reflects the conservation of developmental pathways and cell signaling mechanisms that regulate cardiovascular and renal development, including a central role for cilia in the pathogenesis of CHD and renal birth defects. Together these findings suggest the routine evaluation of CHD patients for renal anomalies with simple non-invasive renal ultrasound may be warranted. This may allow early diagnosis and early therapeutic intervention to treat and manage any renal dysfunction that could negatively impact the long-term outcome of this high-risk patient population.

**Table 2:** Clinical characteristics of congenital heart disease patients.

**ID #**	**Kidney phenotypes**	Cardiac phenotype^*^	**Other phenotypes**
7042	Abnormally rotated kidney, Pelvicaliectasis	HLHS	
7199	Caliectasis (dilation of the renal calices)	HLHS	Hypothyroidism
7351	Duplicated collecting duct system, Hydronephrosis	HLHS	
7208	Duplicated collecting system, Vesicoureteral reflux	D-TGA	
7319	Ectopic kidney	DORV, subaortic VSD	
7040	Ectopic kidney, vesicoureteral reflux	ASD, VSD	
7194	Horseshoe kidney	TOF, PA	
7035	Hydronephrosis	ASD, VSD	Tethered cord, hypothyroidism
7288	Mild Hydronephrosis	Truncus arteriosus	
7053	Pelvicaliectasis	D-TGA	
7302	Pelvicaliectasis	HLHS	
7306	Pelvicaliectasis	D-TGA	
7334	Pelvicaliectasis	ASD, VSD, PDA, Interrupted IVC	
7389	Pelvicaliectasis	HLHS	
7417	Pelvicaliectasis	VSD/PDA/ventricular hypertrophy	
7419	Pelvicaliectasis	DORV, PA, complete AVSD, supracardiac TAPVR	Heterotaxy, asplenia
7430	Pelvicaliectasis	Unbalanced AVSD, PA, supracardiac TAPVR	Heterotaxy, asplenia
7058	Pelvicaliectasis, Hydroureter	TOF	Tethered cord, exotropia, neurogenic bladder
7336	Pyelectasis (dilation of the renal pelvis)	D-TGA	
7289	Renal size asymmetry, Solitary cyst seen by ultrasound.	HLHS	
7474	Unilateral Agenesis	HLHS	Single testis
7027	Vesicoureteral reflux	D-TGA	Situs inversus totalis, seizures
7438	Vesicoureteral reflux	HLHS	Aspiration, hypospadias

* *D-TGA, D-transposition of the great arteries; DORV, double outlet right ventricle; ASD, atrial septal defect; VSD, ventricular septal defect; HLHS, hypoplastic left heart syndrome; TOF, Tetralogy of Fallot; AVSD, atrioventricular septal defect; PA, pulmonary atresia; TAPVR, total anomalous pulmonary venous return. Adapted from San Agustin et al. (10)*.

## Conclusion

Studies in both CHD mutant mice and CHD patients showed CAKUT is highly associated with CHD. This is supported by the finding of CAKUT and CHD in various genetic syndromes, and the finding that many mutations identified to cause CHD can also cause CAKUT phenotypes. Among genes identified to cause both CHD and CAKUT there is a significant enrichment of cilia genes, indicating a central role for cilia in the pathogenesis of CHD and CAKUT phenotypes. However, as half of the mutations identified to cause CHD and renal anomalies are not known to be cilia-related, the association of CHD with renal anomalies likely extend beyond the role of cilia in heart and kidney development. The clinical relevance of these findings in mice were shown with the observation that 23–30% of CHD patients also exhibited renal anomalies. This would suggest CHD patients may benefit from routine evaluation for renal anomalies. This might improve the long-term outcome of this high-risk patient population by reducing potential renal complications with early diagnosis and therapeutic intervention.

## Author contributions

All authors listed contributed intellectually to this work and approved it for publication.

### Conflict of interest statement

The authors declare that the research was conducted in the absence of any commercial or financial relationships that could be construed as a potential conflict of interest.
